# High Prevalence of *Plasmodium falciparum* HRP2/3 Gene Deletions in Ethiopia: Implications for Malaria Diagnosis and Treatment—A Systematic Review and Meta-Analysis

**DOI:** 10.1155/cjid/8677211

**Published:** 2025-10-17

**Authors:** Habtamu Gebrie, Aberham Abere, Anteneh Gashaw, Behailu Taye Gebremeskele, Yeaynmarnesh Asmare Bukayaw, Alem Bayable, Biazin Yenealem Mekuriaw, Eyob Getaneh Yimer, Abel Desalegn Demeke

**Affiliations:** ^1^Department of Medical Laboratory Science, College of Medicine and Health Sciences, Dilla University, P.O. Box 419, Dilla, Ethiopia; ^2^Department of Medical Parasitology, School of Biomedical and Laboratory Science, College of Medicine and Health Science, University of Gondar, P.O. Box 196, Gondar, Ethiopia; ^3^Department of Midwifery, College of Medicine and Health Sciences, Dilla University, P.O. Box 419, Dilla, Ethiopia; ^4^Department of Epidemiology and Biostatistics, Institute of Public Health, College of Medicine and Health Science, University of Gondar, P.O. Box 196, Gondar, Ethiopia; ^5^Department of Nursing, College of Medicine and Health Science, Dilla University, P.O. Box 419, Dilla, Ethiopia; ^6^Department of Psychiatry, College of Health and Medical Science, Dilla University, P.O. Box 419, Dilla, Ethiopia; ^7^School of Medicine, College of Medicine and Health Sciences, Gondar University, P.O. Box 196, Gondar, Ethiopia

**Keywords:** Ethiopia, gene deletions, histidine-rich protein, *Plasmodium falciparum*

## Abstract

**Introduction:**

*Plasmodium* parasite species are the causative agents of malaria, which affects populations worldwide. Rapid diagnostic tests (RDTs), microscopy, and molecular methods have been used to diagnose the disease. HRP2 antigens are unique to *P. falciparum*, while RDTs can detect lactate dehydrogenase, aldolase, and HRP3. Nevertheless, PfHRP2-based RDTs may produce false-negative results if the parasite's PfHRP2/3 genes are deleted. This study is necessary because there is currently no compiled evidence-based information regarding PfHRP2/3 gene deletion in Ethiopia.

**Methods:**

Primary research articles on PfHRP2/3 gene deletions (2000–2025) were retrieved from PubMed, Science Direct, and Google Scholar. Eligible studies were systematically searched between May 3 2025, and June 2, 2025. The quality of the included studies was assessed using the Newcastle–Ottawa Scale. Data analysis was performed using STATA Version 17, employing a random effects model. Heterogeneity among studies was evaluated using the *I*^2^ test. To assess publication bias, Begg's and Egger's tests were conducted along with funnel plot symmetry analysis.

**Results:**

A total of 932 studies were initially identified, among these 18 studies were selected for full-text review. After excluding 7 studies, 11 articles were included in the meta-analysis. The overall pooled prevalence of PfHRP2/3 gene deletions was 35.64% (95% CI: 21.43, 49.85). Specifically, the pooled prevalence of PfHRP2 and PfHRP3 gene deletions was 8.48% (95% CI: 0.96, 16.01) and 23.74% (95% CI: 12.16, 35.32), respectively, while concurrent deletions of both genes account for 8.14% (95% CI: 0.67, 15.61).

**Conclusion:**

This systematic review and meta-analysis revealed a high prevalence of *PfHRP2*/3 gene deletions, highlighting significant challenges to the continued use of PfHRP2/3-based RDTs in malaria control programs in Ethiopia. Further nationwide surveillance using standardized methodologies is recommended to better understand the extent of these gene deletions and to guide the immediate phasing out of PfHRP2/3-based RDTs from the national malaria diagnostic algorithm. We also recommend that PCR be considered an essential diagnostic tool in in-vitro diagnosis (IVD).

## 1. Background

*Plasmodium* parasites species are the causative agent for malaria, and a disease is endemic in over 83 countries worldwide. Globally, 263 million cases and 597, 000 deaths have been reported [1, 2]. In Ethiopia, around 69% of the population is at risk of malaria infection, and over 7.3 million malaria cases and 1157 deaths were reported in 2024; of these, 20% were children under five years of age [3, 4]. Microscopy, rapid diagnostic tests (RDTs), and other molecular methods such as immunoassay, enzyme-linked immunosorbent assay (ELISA), and polymerase chain reaction (PCR) have used to diagnose the disease [5]. RDTs paly an essential contribution for managing malaria cases, while microscopy is a gold standard diagnostic method. RDTs were initially created in 1990 and are now commonly utilized in many malaria-endemic regions because it is affordable, requires minimal training, and yields results in a few minutes [6].

Lactate dehydrogenase (LDH), aldolase, and histidine-rich protein-2/3 (HRP2/3) are the three malaria antigens that RDTs may detect. At the sexual and asexual stages of blood stage malaria, HRP2/3 antigens only express on *Plasmodium falciparum (P. falciparum)* species, making them species-specific [7–12]. However, a variety of variables, including parasite density, the quantity of parasite antigen produced (which stays in peripheral blood), and the variance of target epitopes in antigen structure, could cause PfHRP2-based RDTs to produce false-negative results [13–18]. Additionally, HRP 2/3 gene deletion is linked to the following factors: monoclonal infections [19], individual age [20], the kind of infected individual symptoms [21], and the prevalence of public use of antimalarial medications [22, 23].

HRP2/3 gene deletions first appeared in 2010 [24] and have since been reported in a number of other countries, including India [25], Mali [21], Peru [26], and ten African [27] which can result in false-negative results. In Ethiopia, studies have also reported false-negative HRP2- and HRP3-based RDT results for *Plasmodium* infection [28, 29]. The high rate of false-negative RDT results has led to changes in official diagnostic recommendations in countries [30, 31].

In order to decide whether to alter the national diagnostic guideline policy, it is imperative that the prevalence of PfHRP 2 and 3 gene deletions be regularly monitored, as recommended by the World Health Organization (WHO). Furthermore, if the predicted incidence of *P. falciparum* infections with false-negative HRP2-based RDT results because of PfHRP2/3 deletions is above 5%, WHO also advises using non-PfHRP2-based RDTs. However, governments should set up a regular monitoring program if the projected prevalence is less than 5%. If the 95% CI does not contain 5%, investigations should be repeated in 2 years; if it does, they should be repeated within a year [32]. There is a lack of compiled, evidence-based scientific data regarding the frequency of PfHRP 2 and 3 gene deletions in Ethiopia; this systematic review and meta-analysis is expected to fill this knowledge gap.

## 2. Methods

### 2.1. Study Design

The Preferred Reporting Items for Systematic Reviews and Meta-Analyses (PRISMA) criteria were followed in conducting the systematic review and meta-analysis [33], and the registration number that was assigned to it was CRD42024609542.

### 2.2. Eligibility Criteria

The population intervention comparator outcome (PICO) criteria were used to characterize the study problem. However, since this study was carried out by combining cross-sectional studies, there were neither comparators nor interventions. English-language publications on PfHRP 2 and 3 gene deletion that were undertaken in Ethiopia between January 1, 2000, and June 02, 2025, were included. The search for eligible studies was carried out between May 3, 2025, and June 2, 2025. Observational studies that detailed the frequency of PfHRP2/3 gene deletions in *P. falciparum*-infected people were taken into consideration. Studies that only contained a single participant and were preprint studies were excluded because they had not reported intriguing findings.

### 2.3. Search Strategy

For primary research on PfHRP 2/3 gene deletion, the databases PubMed, Science Direct, and Google Scholar were searched. “Gene deletion” OR “Genetic variability” OR “Protein structure” AND “Pfhrp2/3” OR “*P. falciparum* histidine rich protein 2” OR “*P. falciparum* histidine rich protein 3” AND “Ethiopia”; Filters: Free full text, English, from January 1, 2000, and June 02, 2025 were terms used to search articles. Citations were arranged, and article duplication was checked using the Endnote 21 software.

### 2.4. Risk of Bias Assessment

The Ottawa–Newcastle Scale, which was adjusted for the cross-sectional analysis, was used to evaluate the quality of the included studies [34]. Using the previously indicated tools, H.G., A.A., A.G., B.T.G., Y.A.B., A.B., B.Y.M., E.G.Y., and A.D.D. independently assessed the studies. Selection criteria, comparability, and the methodology for determining study outcomes were taken into account while evaluating studies. Studies with at least an eight on the Ottawa–Newcastle Scale were included in this systematic review and meta-analysis.

### 2.5. Effect Measures

We assessed the overall frequency of PfHRP 2/3 gene deletions in this systematic review and meta-analysis, and the pooled effect was expressed using the odds ratio (OR).

### 2.6. Selection of Studies

In a distinct and indistinguishable way, H.G., A.A., A.G., B.T.G., Y.A.B., A.B., B.Y.M., E.G.Y., and A.D.D. independently assessed the eligibility of the researches based on the inclusion and exclusion criteria. Any disagreements that appeared during the study selection process were discussed and re-examined by the authors, B.T.G. and H.G.

### 2.7. Data Extraction

Using a standard Microsoft Excel spreadsheet, H.G., A.A., A.G., B.T.G., Y.A.B., A.B., B.Y.M., E.G.Y., and A.D.D. separately extracted all the necessary data. Name of the author, year of publication, nation, location, study design, sample type, sample size, overall prevalence of PfHRP2/3, HRP2 prevalence, HRP3 prevalence, and prevalence of both HRP2 and 3 were extracted from each articles, and the primary data extraction arrangements were based on the quality score of the research. Publication year category and sample size category were also used for subgroup analysis.

### 2.8. Synthesis Methods

The analysis was conducted using STATA Version 17. The *I*^2^ test was used to assess heterogeneity in this investigation; a result of 50%, 50%–75%, or > 75% was categorized as low, moderate, or high heterogeneity, respectively [35]. Because of the large degree of variability, a random effects model was employed for analysis. For each primary article, the standard error was calculated using the binomial distribution formula. To identify the source of heterogeneity, subgroup analysis was employed [36–38]. The crude proportion was calculated by dividing the total number of gene deletions (HRP2, HRP3, and both HRP2 and HRP3) by the total number of confirmed *Plasmodium falciparum* samples that were negative with pfHRP2-/pfHRP3-based RDT, which represents the proportion of total gene deletions among the genotyped, *P. falciparum*-confirmed samples. The results of the systematic review and meta-analysis were presented using texts, tables, and forest plots.

### 2.9. Reporting Bias Assessments

To check for publication bias, funnel plots [39] and Begg's and Egger's statistical tests [40] were employed. When the *p*-value was less than 0.05, publication bias was considered to be present.

## 3. Results

### 3.1. Study Search and Selection

**Figure 1:**
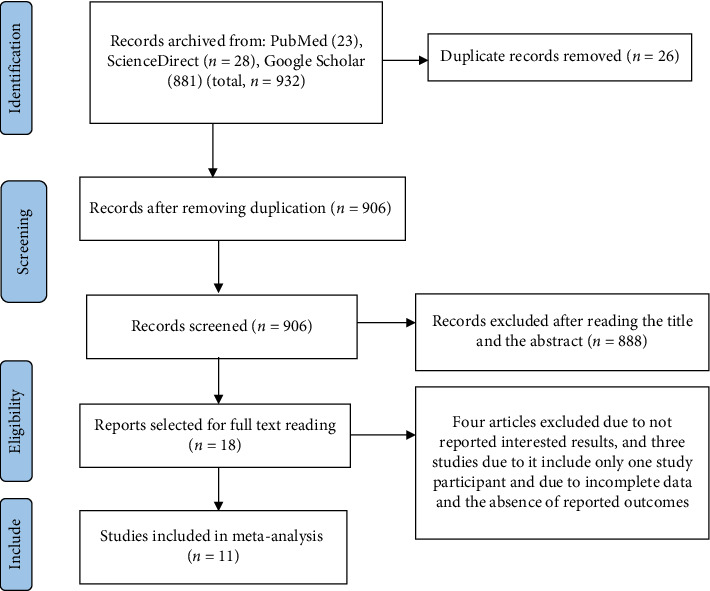
PRISMA flowchart of the selection steps of included studies.

A total of 932 primary studies were initially retrieved from databases. Of these, 26 studies were excluded due to duplication, and 888 were removed based on their titles and abstracts. Eighteen studies were selected for full-text review; however, an additional seven studies were excluded at this stage [41–46], because they did not report relevant outcomes or included only a single study participant. Finally, a total of 11 articles that met the inclusion criteria were included in the meta-analysis [28, 29, 47–56] (Figure 1).

### 3.2. Study Characteristics

This systematic review and meta-analysis were conducted on 30,719 blood samples collected using filter paper (dried blood spots, DBS) from malaria suspected patients. Of these, 2487 samples were genotyped to identify pfHRP2 and pfHRP3 gene deletions and confirm the presence of *Plasmodium falciparum*. The remaining 28,232 blood samples were excluded due to the absence of *Plasmodium* species, the presence of species other than *P. falciparum*, incomplete patient data on DBS, or substandard DBS quality. A total of 11 primary studies were included in the analysis. Ten of these were cross-sectional studies, while one was a therapeutic efficacy study. Among the included studies, seven were conducted in Ethiopia, and four were global studies that also included Ethiopian data. Specifically studies which were conducted in Ethiopia indicated that three studies were carried out in the northern and western regions of Ethiopia, two in the north and east, one in the south, and one in the northwest (Table 1).

The diagnostic methods used across these studies included RDTs, nested PCR (nPCR), quantitative PCR (qPCR), photo-induced electron transfer PCR (PET-PCR), and a multiplex bead-based antigen detection immunoassay (BBI) were used to diagnose *Plasmodium* species and detect pfHRP2/3 gene deletions.

### 3.3. Proportion of PfHRP2 Gene Deletions Among PfHRP2-Based RDT False Negatives

A total of 11 studies were included to estimate the pooled proportion of PfHRP2 gene deletions among PfHRP2-based RDT false-negative cases. The crude analysis showed that the proportion of PfHRP2 deletions among false-negative cases ranged from 4.0% to 80.9%. Using meta-analysis, the estimated proportions across the studies were ranged from 0.14% to 81.00%. The overall pooled proportion of PfHRP2 gene deletions was estimated at 32.57% (95% CI: 17.97, 52.17) (Table 2).

### 3.4. Pooled Prevalence of PfHRP2/3, PfHRP 2, and PfHRP 3 Gene Deletions

**Figure 2:**
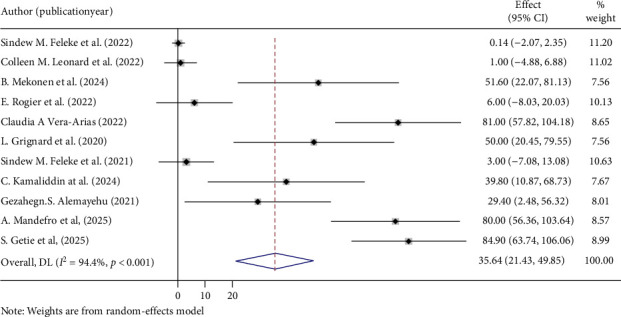
Overall pooled prevalence of PfHRP 2 /3 genes deletions.

**Figure 3:**
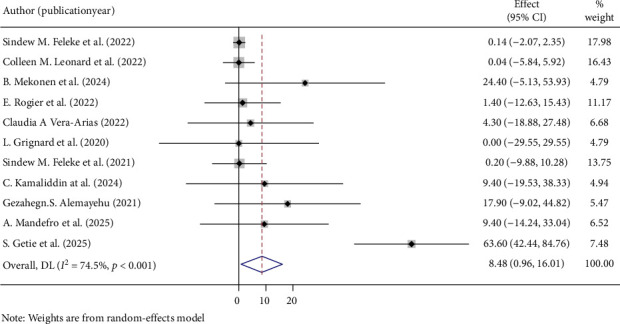
Pooled prevalence of PfHRP 2 genes deletions.

**Figure 4:**
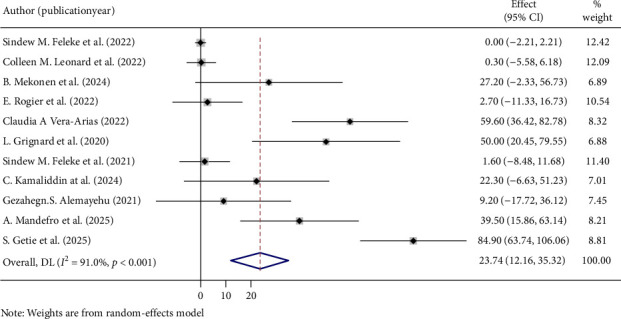
Pooled prevalence of PfHRP 3 genes deletions.

**Figure 5:**
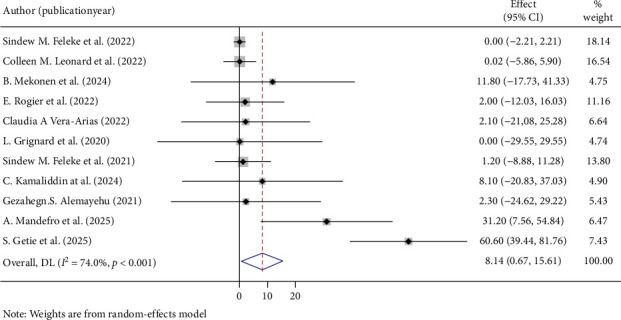
Pooled prevalence of both PfHRP 2 and 3 genes deletions.

The overall pooled prevalence of *PfHRP2/3* gene deletions in this study was 35.64% (95% CI: 21.43, 49.85), with a high level of heterogeneity (*I*^2^ = 94.4%, *p* < 0.001) (Figure 2). Specifically, the pooled prevalence of *PfHRP2* gene deletions was 8.48% (95% CI: 0.96, 16.01) (Figure 3), and for *PfHRP3* deletions, it was 23.74% (95% CI: 12.16, 35.32) (Figure 4). Moreover, the prevalence of samples with deletions in both *PfHRP2* and *PfHRP3* genes was 8.14% (95% CI: 0.67, 15.61) (Figure 5).

### 3.5. Heterogeneity Test for Overall Prevalence of PfHRP2/3

**Figure 6:**
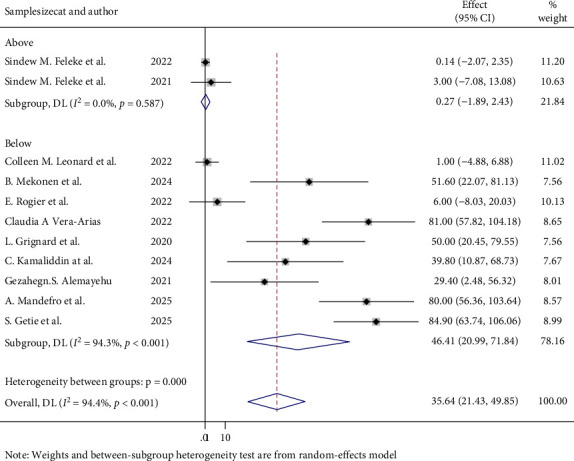
Subgroup analysis between studies based on sample size category.

**Figure 7:**
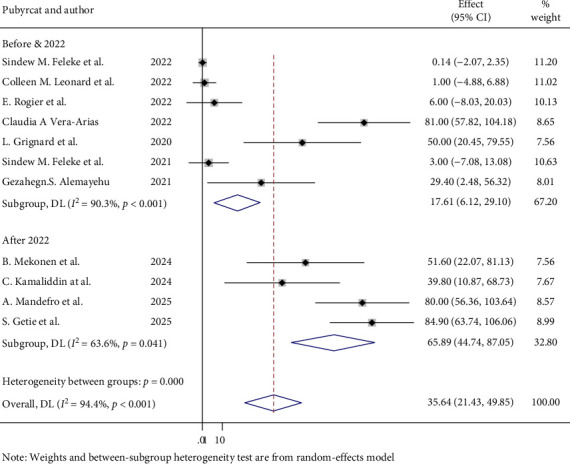
Subgroup analysis between studies based on publication year category.

The heterogeneity between articles was high, *I*^2^ = 94.4%, with a *p*-value of < 0.001. To determine the source of heterogeneity, a subgroup analysis was done for overall prevalence of PfHRP 2/3 gene deletion according to sample size and publication year category. Studies with a sample size below the mean (2,833.6) showed a higher prevalence of PfHRP2/3 gene deletions (46.41%; 95% CI: 20.99–71.84) and greater heterogeneity (*I*^2^ = 94.3%, *p* < 0.001) compared to studies with a sample size above the mean (Figure 6). Furthermore, a subgroup analysis was also done according to publication year category, and there was a high magnitude of PfHRP 2/3 gene deletion in studies that were conducted after 2022 [65.89%; 95% CI (44.74, 87.05)] and moderate heterogeneity (63.6%; *p* = 0.41) than the study conducted before 2022 (Figure 7). Although several included studies did not report the specific locations of sample collection area, which limits the feasibility of a subgroup analysis by geographical location, the observed high heterogeneity might be influenced by geographical variations.

### 3.6. Publication Bias Between Studies and Sensitivity Test for Overall Prevalence of PfHRP2/3

**Figure 8:**
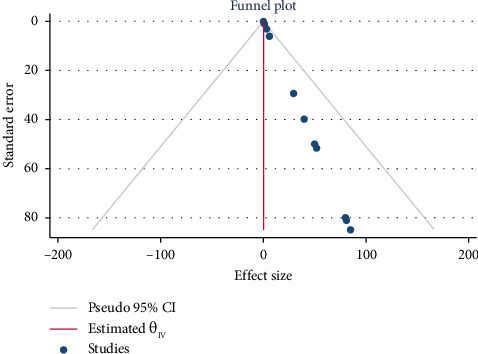
Pfhrp 2/3 overall pooled prevalence estimation using a funnel plot.

**Figure 9:**
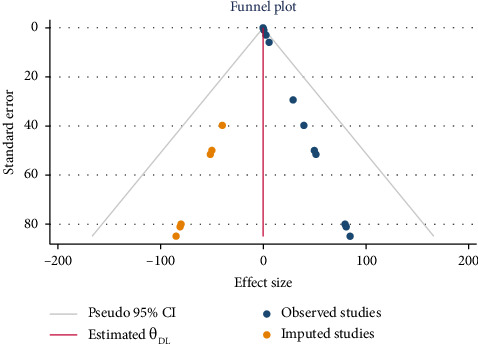
Trim-and-fill analysis for adjusting effect size.

There was publication bias in this systematic review and meta-analysis, as confirmed by asymmetric funnel plot (Figure 8), and a Begg's and Egger test indicates that there is a publication bias (*p*=0.002). Therefore, in order to know the effect of small effect size and unpublished studies on the overall effect size of the meta-analysis, trim-and-fill analysis was conducted. As indicted in Figure 9, after using trim-and-fill analysis, the plot became more symmetric, and the pooled effect estimate decreased. This implies that the original estimate was probably overestimated due to missing negative/smaller studies, which is a hallmark of publication bias.

**Figure 10:**
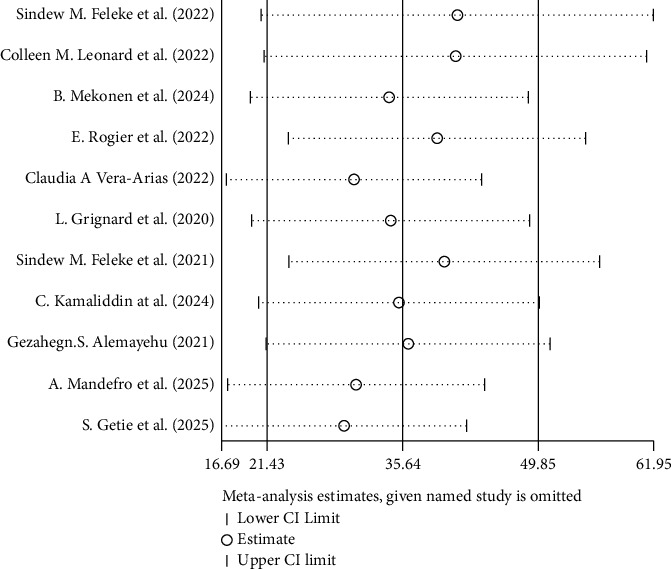
Sensitivity test for the estimation of overall pooled prevalence of PfHRP2/3.

A sensitivity analysis employing the leave-one-out method was also done to investigate the impact of a single study on the entire meta-analysis. As can be seen in Figure 10, the findings provide no convincing proof of how one study affected the overall meta-analysis result.

## 4. Discussion

A study done globally noted that PfHRP2 and 3 gene deletion was observed in many nations [57]. Ethiopia is one of the malaria-endemic countries [2] where the population is frequently exposed to malaria infection. Studies conducted in Ethiopia report that over 90% of cases had a history of previous malaria exposure and antimalarial drug use, and more than half of the participants were within the highly vulnerable age group of less than 15 years [58, 59]. Consequently, host-related factors, parasite species, and prior antimalarial drug use are key contributors to diagnostic challenges and the emergence of drug resistance [60, 61].

The overall pooled prevalence of current study was higher than the systematic review and meta-analysis conducted globally 21.30% [57], and the study was carried out in Cameroon 2.2% [62], in Africa 4%, and in Asia 3% [63] This discrepancy might be due to human, environmental, or parasitic natural selection [60, 61]; moreover, it might also be due the frequency of antimalarial drug use by the general public (frequently treated individuals are highly associated with gene deletion) [19], individual age (younger age more vulnerable for gene deletion) [20], and the type of symptoms experienced by the infected person (deletions are higher for asymptomatic individuals) [21]. Moreover, the current study in line with the studies was conducted in Africa 7% and India 5% [64]. However, in the same reason that mentioned above, the pooled prevalence of PfHRP2 gene deletion in this study was lower than the study conducted in South–Central America 18% [63].

The PfHRP3 gene encodes a protein capable of cross-reacting with the monoclonal antibodies used in PfHRP2-based RDTs, thereby potentially reducing false-negative results. However, this review also identified a high prevalence of PfHRP3 gene deletions. The reported deletion rates across individual studies ranged from 0.0% to 84.9%, with a pooled overall prevalence of 23.74% [64].

This study examined PfHRP2 gene deletions among false-negative results from PfHRP2-based RDTs, with deletion rates ranging from 4.0% to 80.9% and a pooled proportion of 32.57% (17.97, 52.17). The high rate of deletions compromises the accuracy of these RDTs, increasing the risk of misdiagnosis and inappropriate treatment for other infections with similar symptoms [65].

There was substantial heterogeneity among the studies included in this analysis. Subgroup analysis indicated that the heterogeneity of the included studies was primarily due to differences in sample size and publication year across the studies. Additionally, the evidence of publication bias was found. This implies that the original estimate was probably overestimated due to missing negative/smaller studies, which is a hallmark of publication bias. A sensitivity analysis, using the leave-one-out method, was also performed to assess the influence of individual studies on the overall meta-analysis results [66]. Therefore, the results of the current study did not provide compelling evidence about the impact of one study on the overall meta-analysis result.

### 4.1. Limitation of the Study

The limitation of this study is the inability to conduct a comprehensive exploration of the sources of heterogeneity. This was primarily due to the lack of essential information in several of the included studies. For instance, many studies did not report the specific geographical locations of their sample collection sites within Ethiopia. Without such detailed information, it was not possible to perform subgroup analyses based on geographical regions, which could have provided valuable insights into regional variations and potential contributors to heterogeneity.

## 5. Conclusion and Recommendation

This systematic review and meta-analysis revealed a high prevalence of *PfHRP2*/3 gene deletions, highlighting significant challenges to the continued use of HRP2/3-based diagnostic tools in malaria control programs. The findings cast doubt on the reliability of *PfHRP2/3*-based diagnosis for *Plasmodium falciparum* malaria. Therefore, further nationwide surveillance using standardized methodologies is recommended to better understand the extent of these gene deletions and to guide the immediate phasing out of PfHRP2-/3-based RDTs from the national malaria diagnostic algorithm. We also recommend that PCR be considered an essential diagnostic tool in in-vitro diagnosis.

## Figures and Tables

**Table 1:** Characteristics of studies included in the systematic review and meta-analysis.

Author name	Pub year	Location	Included sample for genotyping	Type diagnostic methods	Pf confirmation	Methods of pfHRP2 gene deletion detection
Sindew M. Feleke et al.	2022	North & west	13,172	RDTs (SD-Bio line, PLDH, CareStart), PCR, BBI	BBI/PCR	PCR
Colleen M. Leonard et al.	2022	North & east	2648	RDT, BBI	BBI	BBI
B. Mekonen et al.	2024	South	279	Microscopy, RDT (SD-Bio line)	nPCR	nPCR
E. Rogier et al.	2022	Not stated	147	Photo-induced electron transfer PCR, RDTs, nPCR	Photo induced electron transfer PCR	nPCR
Claudia A Vera-Arias et al.	2022	Not stated	294	ddPCR, qPCR	ddPCR,	ddPCR
L. Grignard et al.	2020	Not stated	47	RDT, qPCR	qPCR	qPCR
Sindew M. Feleke et al.	2021	North & west	2	RDT, qPCR, BBI. Assay	qPCR, MIP	qPCR
C. Kamaliddin et al.	2024	Not stated	12,572	RDT (SD Bioline), ddPCR	ddPCR	ddPCR
Gezahegn S. Alemayehu et al.	2021	North & west	233	RDT (PfHRP2), microscopy, qPCR	qPCR	qPCR
S. Getie et al.	2025	Northwest	302	nPCR	nPCR	nPCR
A. Mandefro et al.	2025	Northwest	1023	RDTs, nPCR, microscopy	nPCR	nPCR
Combined	—	Ethiopia	30,719	—	—	—

*Note:* Pub year = publication year.

Abbreviations: BBI = bead-based immunoassay, hrp2 = histidine-rich protein 2, hrp3 = histidine-rich protein 3, PCR = polymerase chain reaction.

**Table 2:** Estimated proportions of pfHRP2/3 gene deletions among false-negative cases using PfHRP2-based RDTs.

Author name	Pub year	Location	# confirmed PF, but negative pfHRP2-based RDT	Number and proportion of pfHRP2-/3-using crude analysis	Estimate (95% CI) using meta-analysis
Sindew M. Feleke et al.	2022	North & west	456	19 (4)	0.14 (2.07, 2.35)
Colleen M. Leonard et al.	2022	North & east	29	15 (52)	1.00 (4.88, 6.88)
B. Mekonen et al.	2024	South	249	144 (57.8)	51.60 (22.07, 81.03)
E. Rogier et al.	2022	Southwest & northwest	147	9 (6)	6.00 (8.03, 20.03)
Claudia A Vera-Arias et al.	2022	Not stated	47	38 (80.9)	81.00 (57.82, 104.18)
L. Grignard et al.	2020	Not stated	2	1 (50)	50.00 (20.45, 79.55)
Sindew M. Feleke et al.	2021	North & west	610	355 (58)	3.00 (7.08, 13.08)
C. Kamaliddin at al.	2024	Not stated	233	71 (30.5)	39.80 (10.87, 68.73)
Gezahegn S. Alemayehu	2021	North & west	218	64 (29.4)	29.40 (2.48, 56.32)
S. Getie et al.	2025	Northwest	33	21 (63.6)	16.30 (5.53, 38.13)
A. Mandefro et al.	2025	East & northwest	463	44 (9.4)	80.00 (56.36, 103.64)
Combined	—	All of the location	2487	781 (40.15)	32.57 (17.97, 52.17)

## Data Availability

All the data can be accessed from the primary author (Habtamu Gebrie).

## Supporting Information

Supporting Information 1 The quality of the included articles was assessed using a quality appraisal checklist. Among them, four studies scored 9, while the remaining seven studies scored 8 (Supporting File 1).

Supporting Information 2 Additionally, the PRISMA 2020 guideline and the PRISMA checklist were employed to ensure methodological rigor (Supporting File 2).
