# Supplementary material for: High Prevalence of Plasmodium falciparum HRP2/3 Gene Deletions in Ethiopia: Implications for Malaria Diagnosis and Treatment—A Systematic Review and Meta-Analysis
Source: Can J Infect Dis Med Microbiol. 2025 Oct 17;2025:8677211. doi: 10.1155/cjid/8677211 (PMC12552085; doi:10.1155/cjid/8677211)
Supplement: Supporting Information 1 — The quality of the included articles was assessed using a quality appraisal checklist. Among them, four studies scored 9, while the remaining seven studies scored 8 (Supporting File 1). [file 8677211.f1.docx]

| Author, Publication year | Selection: (Maximum 5 stars) | | | | Comparability: Max 1 star | Outcome: (Maximum 3 stars) | | Total |
| --- | --- | --- | --- | --- | --- | --- | --- | --- |
|  | Q 1a | Q 2a | Q 3a | Q4a | Q 1a | Q 1a | Q 2 a |  |
| Sindew M. Feleke et al. | 1 | 1 | 1 | 1 | 1 | 2 | 1 | 8 |
| Colleen M. Leonard et al. | 1 | 1 | 1 | 2 | 1 | 2 | 1 | 9 |
| B. Mekonen et al. | 1 | 1 | 1 | 2 | 1 | 1 | 1 | 8 |
| E. Rogier et al. | 1 | 1 | 1 | 2 | 1 | 1 | 1 | 8 |
| Claudia A Vera-Arias | 1 | 1 | 1 | 1 | 1 | 2 | 1 | 8 |
| L. Grignard et al. | 1 | 1 | 1 | 1 | 1 | 2 | 1 | 8 |
| Sindew M. Feleke et al. | 1 | 1 | 1 | 2 | 1 | 2 | 1 | 9 |
| C. Kamaliddin at al. | 1 | 1 | 1 | 1 | 1 | 2 | 1 | 8 |
| Gezahegn.S. Alemayehu | 1 | 1 | 1 | 2 | 1 | 2 | 1 | 9 |
| A.Mandefro et al. | 1 | 1 | 1 | 2 | 1 | 2 | 1 | 9 |
| S. Getie et al. | 1 | 1 | 1 | 1 | 1 | 2 | 1 | 8 |

**Quality appraisal check list**

**NEWCASTLE - OTTAWA QUALITY ASSESSMENT SCALE**

**Selection: (Maximum 5 scores)**

**1. Representativeness of the cases**: **[Q 1 a)** truly representative of the HCC patients (consecutive or random sampling of cases). 1 score], **[Q 1 b)** Some-what representative of the average in the HCC patients (non-random sampling).1 score], **[Q 1 c)** Selected demographic group of users. 0 score], **[Q 1 d)** No description of the sampling strategy. 0 score], **2. Sample size**: [Q 2 a) Justified and satisfactory (≥ 400 HCC included) 1 score], **[Q 2 b)** Not justified (<400 HCC patients included). 0 score], **3. Non-Response rate: [Q 3 a**) The response rate is satisfactory (≥95%).1 Score], [**Q3 b**) The response rate is unsatisfactory (<95%), or no description. 0 Score], **4. Ascertainment of the screening/surveillance tool: [Q 4 a**) validated screening/surveillance tool. 2 scores], **[Q 4 b)** Non-validated screening/surveillance tool, but the tool is available or described. 1 score],**[Q 4 c)** No description of the measurement tool. 0 score].

**Comparability: (Maximum 1 stars)**

1) **The potential confounders were investigated by subgroup analysis or multivariable analysis. [Q 1a)** the study investigates potential confounders. 1 score], **[Q1 b**) the study does not investigate potential confounders. 0 score].

**Outcome: (Maximum 3 stars)**

**1. Assessment of the outcome:** **[Q 1 a)** Independent blind assessment. 2 scores], **[Q 1 b)** record linkage. 2 scores], **[Q 1 c)** Self-report. 1 score], **[Q1 d)** No description. 0 score], 2**. Statistical test:** **[Q 2 a)** the statistical test used to analyze the data is clearly described and appropriate. 1 score], **[Q2 b)** the statistical test is not appropriate, not described or incomplete. 0 score]
